# Feasibility and Acceptability of App-Based Mindfulness-Meditation Training for Older Adults

**DOI:** 10.1093/geroni/igaa057.1324

**Published:** 2020-12-16

**Authors:** Leeann Mahlo, Tim Windsor

**Affiliations:** 1 Flinders University, Woodside, South Australia, South Australia, Australia; 2 Flinders University, Adelaide, South Australia, Australia

## Abstract

Few studies have focused on the utility of mindfulness-meditation for well-being in older adults. This study aimed to investigate the feasibility and acceptability of an app-based mindfulness-meditation program for community-based older adults. A convenience sample of 46 participants aged between 63 and 81 (M = 70.85, SD = 4.70) was recruited from the community. Participants were invited to engage with a 30-day app-based mindfulness-meditation program for 10-minutes daily on their smartphones. Each meditation session comprised focusing on the breath, mentally scanning the body, monitoring the mind’s activity, and cultivating a nonjudgmental attitude toward experience. Participants completed psychosocial questionnaires at baseline, day 10, and day 30. On average, participants completed 25 sessions and almost 4 hours of application use across the 30-days. Results of linear mixed effects models showed significant improvements in positive affect, negative affect, and life satisfaction across the study interval, but no meaningful change in total or facet-level mindfulness or perceived stress. Furthermore, relative to high levels of smartphone efficacy, low smartphone efficacy was associated with higher perceived stress and negative affect, and less life satisfaction at baseline; and steeper improvements on these outcomes across the study interval. Results indicated that, on average, older adults found app-based mindfulness-meditation training interesting, enjoyable, valuable, and useful. The findings provide preliminary support for the feasibility and acceptability of an app-based mindfulness-meditation program with community-based older adults and demonstrate potential benefits for well-being. Furthermore, older adults’ perceptions of smartphone competency may play an important role in the outcomes of app-based programs.

